# Supplementary material for: Reduction in the Migration Activity of Microglia Treated with Silica-Coated Magnetic Nanoparticles and their Recovery Using Citrate
Source: Cells. 2022 Aug 3;11(15):2393. doi: 10.3390/cells11152393 (PMC9368468; doi:10.3390/cells11152393)
Supplement: Supplementary file 1 [file cells-11-02393-s001.zip › cells-1834970-supplementary.pdf]

Supplementary Materials

Reduction in the migration activity of microglia treated with silica-coated-magnetic-nanoparticles and its recovery using citrate

Tae Hwan Shin [1,#], Da Yeon Lee [1,#], Yong Eun Jang [1,#], Do Hyeon Kwon [1], Ji Su Hwang [2], Seok Gi Kim [2], Chan Seo [3], Man Jeong Paik [3], Ju Yeon Lee [4], Jin Young Kim [4], Seokho Park [5], Sung-E Choi [1], Shaherin Basith [1], Myeong Ok Kim [6], Gwang Lee [1,2,*]

[1] Department of Physiology, Ajou University School of Medicine, 206 World Cup-ro, Suwon 16499, Republic of Korea
[2] Department of Molecular Science and Technology, Ajou University, 206 World Cup-ro, Suwon 16499, Republic of Korea
[3] College of Pharmacy, Sunchon National University, 255 Jungang-ro, 57922 Suncheon, Republic of Korea
[4] Research Center of Bioconvergence Analysis, Korea Basic Science Institute, 162 Yeongudanji-ro, 28119 Cheongju, Republic of Korea
[5] Department of Biomedical Science, Graduate School of Ajou University, 206 World Cup-ro, Suwon 16499, Republic of Korea
[6] Division of Life Science and Applied Life Science (BK21 FOUR), College of Natural Sciences, Gyeongsang National University, Jinju 52828, Republic of Korea
[#] These authors contributed equally
[*] Correspondence: glee@ajou.ac.kr

Table S1. Quantitative real-time PCR primer sequences for transcriptomic network-related genes

Gene Name	Symbol	NCBI Ref. seq	Direction	Primer sequence (5'-3')
formyl peptide receptor 1	Fpr1	NM_013521.2	Forward	GACTTGGACCAGGGAAAACA
			Reverse	GGAGTGCTGAACCCAATGAT
protein kinase C, beta	Prkcb	BC048553.1	Forward	GTATCCCAAGTCCATGTCCAAG
			Reverse	TGCTCCTTAATGTCTCGTTCC
serine (or cysteine) SH2 domain protein 2A	Sh2d2a	BC034847.1	Forward	TGAAGCGAGATGGAGTGTTC
			Reverse	GGATGGAGAGTGGTTGAAGG
microtubule-associated protein tau	Mapt	BC014748.1	Forward	GATTGAAACCCACAAGCTGAC
			Reverse	TGGAAGACACATTGCTGAGG
glyceraldehyde-3-phosphate dehydrogenase	Gapdh	NM_001289726.1	Forward	AAATTCAACGGCACAGTCAAG
			Reverse	CTCCACGACATACTCAGCAC

Ref. seq.: Reference sequence

Table S2. Ingenuity pathway analysis-based transcriptome profiles of BV2 cells treated with MNPs@SiO_2(RITC)

Canonical pathways	-log(p-value)
Protein Kinase A Signaling	3.06
Gustation Pathway	3.04
Granulocyte Adhesion and Diapedesis	2.98
GP6 Signaling Pathway	2.88
Altered T Cell and B Cell Signaling in Rheumatoid Arthritis	2.87
Leukocyte Extravasation Signaling	2.75
Cardiac β-adrenergic Signaling	2.71
CCR5 Signaling in Macrophages	2.69
Axonal Guidance Signaling	2.55
Hepatic Fibrosis / Hepatic Stellate Cell Activation	2.44
Eicosanoid Signaling	2.38
NAD biosynthesis II (from tryptophan)	2.1
cAMP-mediated signaling	2.1
Th1 and Th2 Activation Pathway	2.09
Th1 Pathway	2.04
Crosstalk between Dendritic Cells and Natural Killer Cells	1.99
Phagosome Formation	1.82
Complement System	1.82
Neuroprotective Role of THOP1 in Alzheimer's Disease	1.8
Notch Signaling	1.76

Table S3. The top 20 biological functions were analysed algorithmically by ingenuity Pathway analysis in the transcriptome of BV2 cells treated with MNPs@SiO_2(RITC)

Biological functions	p-value
Cancer	1.78×10^{22}
Organismal Injury and Abnormalities	1.78×10^{22}
Dermatological Diseases and Conditions	9.55×10^{21}
Endocrine System Disorders	4.38×10^{17}
Gastrointestinal Disease	1.82×10^{14}
Hematological System Development and Function	4.77×10^{13}
Tissue Morphology	4.77×10^{13}
Cellular Development	1.83×10^{12}
Cellular Growth and Proliferation	1.83×10^{12}
Lymphoid Tissue Structure and Development	1.83×10^{12}
Reproductive System Disease	1.92×10^{11}
Cellular Movement	4.18×10^{11}
Inflammatory Response	1.09×10^{10}
Metabolic Disease	1.12×10^{10}
Cellular Function and Maintenance	1.48×10^{10}
Connective Tissue Disorders	3.3×10^{10}
Inflammatory Disease	3.3×10^{10}
Skeletal and Muscular Disorders	3.3×10^{10}
Immunological Disease	3.42×10^{10}
Hematopoiesis	1.69×10^{9}

Table S4. Ingenuity pathway analysis-based transcriptome profiles of BV2 cells treated with MNPs@SiO_2(RITC)

Entrez gene name	Symbol	Ensembl	Location	Signal fold change[a]	
				0.01 μg/μl	0.1 μg/μl
PPARG coactivator 1 alpha	PPARGC1A	ENSMUSG00000029167	Nucleus	2.00	7.80
microtubule associated protein tau	MAPT	ENSMUSG00000018411	Plasma Membrane	11.29	5.26
SH2 domain containing 2A	SH2D2A	ENSMUSG00000028071	Cytoplasm	1.00	5.00
EPH receptor B2	EPHB2	ENSMUSG00000028664	Plasma Membrane	2.67	3.83
nuclear factor, erythroid 2	NFE2	ENSMUSG00000058794	Nucleus	2.10	3.43
zeta chain of T cell receptor associated protein kinase 70	ZAP70	ENSMUSG00000026117	Plasma Membrane	0.00	3.00
C-X-C motif chemokine ligand 3	CXCL3	ENSMUSG00000058427	Extracellular Space	1.35	2.67
colony stimulating factor 3	CSF3	ENSMUSG00000038067	Extracellular Space	1.20	2.48
PML-RARA regulated adaptor molecule 1	PRAM1	ENSMUSG00000032739	Other	3.00	2.44
C-C motif chemokine ligand 2	CCL2	ENSMUSG00000035352	Extracellular Space	1.38	2.40
integrin subunit beta 3	ITGB3	ENSMUSG00000020689	Plasma Membrane	2.00	2.33
serpin family B member 5	SERPINB5	ENSMUSG00000067006	Extracellular Space	3.24	2.31
C-C motif chemokine receptor 5 (gene/pseudogene)	CCR5	ENSMUSG00000079227	Plasma Membrane	1.27	2.15
hepatocyte nuclear factor 4 alpha	HNF4A	ENSMUSG00000017950	Nucleus	1.67	2.00
protein kinase C theta	PRKCQ	ENSMUSG00000026778	Cytoplasm	1.00	2.00
peroxisome proliferator activated receptor gamma	PPARG	ENSMUSG00000000440	Nucleus	1.09	1.82
glutaminase 2	GLS2	ENSMUSG00000044005	Cytoplasm	1.38	1.79
advanced glycosylation end-product specific receptor	AGER	ENSMUSG00000015452	Plasma Membrane	1.41	1.68
aryl hydrocarbon receptor	AHR	ENSMUSG00000019256	Nucleus	1.56	1.67
protein tyrosine phosphatase non-receptor type 22	PTPN22	ENSMUSG00000027843	Cytoplasm	1.48	1.66
thioredoxin interacting protein	TXNIP	ENSMUSG00000038393	Cytoplasm	1.15	1.65
GATA binding protein 1	GATA1	ENSMUSG00000031162	Nucleus	6.13	1.58
NAD(P)H quinone dehydrogenase 1	NQO1	ENSMUSG00000003849	Cytoplasm	-1.24	1.54
C-type lectin domain containing 7A	CLEC7A	ENSMUSG00000079293	Plasma Membrane	1.03	-1.50
MYC proto-oncogene, bHLH transcription factor	MYC	ENSMUSG00000022346	Nucleus	1.15	-1.51
apolipoprotein C2	APOC2	ENSMUSG00000002992	Extracellular Space	-1.30	-1.52
transgelin	TAGLN	ENSMUSG00000032085	Cytoplasm	1.27	-1.53
Fas cell surface death receptor	FAS	ENSMUSG00000024778	Plasma Membrane	-1.08	-1.56
platelet and endothelial cell adhesion molecule 1	PECAM1	ENSMUSG00000020717	Plasma Membrane	-1.91	-1.58
nuclear factor, erythroid 2 like 3	NFE2L3	ENSMUSG00000029832	Nucleus	-1.78	-1.68
cytochrome b-245 beta chain	CYBB	ENSMUSG0	Cytoplasm	-1.12	-1.70

		0000015340			
lipocalin 2	LCN2	ENSMUSG0 0000026822	Extracellular Space	-1.11	-1.70
matrix metallopeptidase 14	MMP14	ENSMUSG0 0000000957	Extracellular Space	-1.03	-1.71
interferon regulatory factor 6	IRF6	ENSMUSG0 0000026638	Nucleus	-1.50	-1.71
NADH:ubiquinone oxidoreductase subunit S4	NDUFS4	ENSMUSG0 0000021764	Cytoplasm	-1.26	-1.72
formyl peptide receptor 2	FPR2	ENSMUSG0 0000052270	Plasma Membrane	-1.26	-1.92
DNA damage inducible transcript 4	DDIT4	ENSMUSG0 0000020108	Cytoplasm	1.05	-2.03
NADPH oxidase 1	NOX1	ENSMUSG0 0000031257	Cytoplasm	1.38	-2.06
interleukin 10	IL10	ENSMUSG0 0000016529	Extracellular Space	-1.28	-2.11
Pim-2 proto-oncogene, serine/threonine kinase	PIM2	ENSMUSG0 0000031155	Nucleus	-1.01	-2.17
latent transforming growth factor beta binding protein 1	LTBP1	ENSMUSG0 0000001870	Extracellular Space	-1.21	-2.27
integrin subunit alpha L	ITGAL	ENSMUSG0 0000030830	Plasma Membrane	-1.28	-2.31
haptoglobin	HP	ENSMUSG0 0000031722	Extracellular Space	-1.18	-2.69
integrin subunit beta 4	ITGB4	ENSMUSG0 0000020758	Plasma Membrane	-1.13	-3.59
protein kinase C beta	PRKCB	ENSMUSG0 0000052889	Cytoplasm	-4.43	-3.88
formyl peptide receptor 1	FPR1	ENSMUSG0 0000045551	Plasma Membrane	-1.38	-4.06
solute carrier family 1 member 2	SLC1A2	ENSMUSG0 0000005089	Plasma Membrane	-2.74	-4.20
ceruloplasmin	CP	ENSMUSG0 0000003617	Extracellular Space	-2.26	-5.22

[a]Normalised signal fold change of signal in treated groups with MNPs@SiO_2(RITC) to corresponding signal of in control group

Table S5. Ingenuity pathway analysis-based profiles of the proteome of BV2 cells treated with MNPs@SiO_2(RITC)

Canonical pathways	-log(p-value)
Superpathway of Cholesterol Biosynthesis	8.16
Glucocorticoid Receptor Signaling	8.16
LXR/RXR Activation	6.6
Role of Macrophages, Fibroblasts and Endothelial Cells in Rheumatoid Arthritis	5.34
Superpathway of Geranylgeranyldiphosphate Biosynthesis I (via Mevalonate)	5.3
Mevalonate Pathway I	4.38
Aryl Hydrocarbon Receptor Signaling	4.26
Acute Phase Response Signaling	4.11
Role of NFAT in Regulation of the Immune Response	4.07
Phagosome Maturation	4.06
Atherosclerosis Signaling	3.96
Phospholipase C Signaling	3.89
Endoplasmic Reticulum Stress Pathway	3.64
Apelin Endothelial Signaling Pathway	3.5
Role of PKR in Interferon Induction and Antiviral Response	3.45
Cholecystokinin/Gastrin-mediated Signaling	3.4
Estrogen Receptor Signaling	3.39
Hepatic Fibrosis Signaling Pathway	3.38
iNOS Signaling	3.29
LPS/IL-1 Mediated Inhibition of RXR Function	3.27

Table S6. Top 20 biological functions evaluated algorithmically by Ingenuity Pathway Analysis in the proteome of BV2 cells treated with MNPs@SiO_2(RITC)

Biological functions	p-value
Organismal Survival	1.98×10^{20}
Cell Death and Survival	4.22×10^{16}
Organismal Development	4.02×10^{15}
Cardiovascular System Development and Function	3.49×10^{14}
Inflammatory Response	7.97×10^{14}
Organismal Injury and Abnormalities	7.97×10^{14}
Tissue Morphology	1.01×10^{13}
Cellular Compromise	2.22×10^{13}
Infectious Diseases	5.5×10^{13}
Cancer	6.06×10^{13}
Reproductive System Disease	6.06×10^{13}
Cellular Movement	1.44×10^{12}
Embryonic Development	3.3×10^{12}
Hematological System Development and Function	3.68×10^{12}
Tumor Morphology	1.58×10^{11}
Cellular Development	1.59×10^{11}
Cellular Growth and Proliferation	1.59×10^{11}
Lymphoid Tissue Structure and Development	1.59×10^{11}
Cell-To-Cell Signaling and Interaction	1.78×10^{11}
Cellular Function and Maintenance	3.19×10^{11}

Table S7. Ingenuity pathway analysis-based profiles of the proteome of BV2 cells treated with MNPs@SiO_2(RITC)

Entrez gene name	Symbol	UniProt	Location	Signal fold change[a]	
				0.01 μg/μl	0.1 μg/μl
POU class 2 homeobox 1	POU2F1	P25425	Nucleus	7.60	5.73
CD80 molecule	CD80	Q00609	Plasma Membrane	2.11	5.43
apolipoprotein A1	APOA1	Q00623	Extracellular Space	9.78	5.11
CD14 molecule	CD14	P10810	Plasma Membrane	2.15	4.95
TNF receptor superfamily member 1B	TNFRSF1B	P25119	Plasma Membrane	-2.04	4.52
myocyte enhancer factor 2D	MEF2D	Q63943	Nucleus	2.43	3.77
CD274 molecule	CD274	Q9EP73	Plasma Membrane	-2.33	3.42
CCAAT enhancer binding protein beta	CEBPB	P28033	Nucleus	-7.26	3.35
keratin 8	KRT8	P11679	Cytoplasm	-3.72	3.17
cathepsin B	CTSB	P10605	Cytoplasm	1.96	3.07
Jun proto-oncogene, AP-1 transcription factor subunit	JUN	P05627	Nucleus	2.23	3.02
tenascin C	TNC	Q80YX1	Extracellular Space	3.54	3.00
thrombospondin 1	THBS1	P35441	Extracellular Space	3.08	2.94
alpha-2-macroglobulin	A2M	Q6GQT1	Extracellular Space	6.23	2.93
ADAM metallopeptidase with thrombospondin type 1 motif 1	ADAMTS1	P97857	Extracellular Space	-2.76	2.85
pleckstrin homology domain containing O1	PLEKHO1	Q9JIY0	Plasma Membrane	2.31	2.79
galectin 9B	LGALS9B	O08573	Cytoplasm	2.64	2.73
glutamate-ammonia ligase	GLUL	P15105	Cytoplasm	2.52	2.65
ATPase H+ transporting accessory protein 2	ATP6AP2	Q9CYN9	Cytoplasm	-1.97	2.65
activated leukocyte cell adhesion molecule	ALCAM	Q61490	Plasma Membrane	2.04	2.57
plexin D1	PLXND1	Q3UH93	Plasma Membrane	-8.78	2.54
keratin 6B	KRT6B	P50446	Cytoplasm	-7.09	2.53
zinc finger and BTB domain containing 7A	ZBTB7A	O88939	Nucleus	2.46	2.48
laminin subunit gamma 1	LAMC1	P02468	Extracellular Space	1.58	2.43
desmoplakin	DSP	E9Q557	Plasma Membrane	-3.46	2.42
coagulation factor VII	F7	P70375	Plasma Membrane	3.91	2.31
vasoactive intestinal peptide	VIP	P32648	Extracellular Space	-2.88	2.31
sphingosine-1-phosphate receptor 2	S1PR2	P52592	Plasma Membrane	-10.27	2.28
keratin 16	KRT16	Q9Z2K1	Cytoplasm	-2.59	2.24
folliculin interacting protein 1	FNIP1	Q68FD7	Cytoplasm	1.66	2.22
transcription factor CP2	TFCP2	Q9ERA0	Nucleus	2.04	2.02
albumin	ALB	P07724	Extracellular Space	4.27	1.90
methyl-CpG binding protein 2	MECP2	Q9Z2D6	Nucleus	2.35	1.55
BRCA2 DNA repair associated	BRCA2	P97929	Nucleus	2.35	1.54
C-terminal binding protein 2	CTBP2	P56546	Nucleus	-2.24	-1.55
sorting nexin 17	SNX17	Q8BVL3	Cytoplasm	-3.14	-1.55
phosphodiesterase 4D interacting protein	PDE4DIP	Q80YT7	Cytoplasm	-2.02	-1.55
ADP ribosylation factor 1	ARF1	P84078	Cytoplasm	-2.51	-1.57
intercellular adhesion molecule 1	ICAM1	P13597	Plasma Membrane	-3.38	-1.63
Fas cell surface death receptor	FAS	P25446	Plasma	-3.73	-1.76

			Membrane		
nuclear factor kappa B subunit 1	NFKB1	P25799	Nucleus	-3.36	-1.81
enolase 1	ENO1	P17182	Cytoplasm	-2.18	-1.89
cyclin dependent kinase 1	CDK1	P11440	Nucleus	-7.91	-2.02
SRC proto-oncogene, non-receptor tyrosine kinase	SRC	P05480	Cytoplasm	-2.47	-2.11
cyclin dependent kinase 5	CDK5	P49615	Nucleus	-2.39	-2.31
serum amyloid A 3	Saa3	P04918	Extracellular Space	-2.58	-2.41
ATPase H+ transporting V1 subunit C1	ATP6V1C1	Q9Z1G3	Cytoplasm	-3.06	-2.71
stathmin 1	STMN1	P54227	Cytoplasm	-2.31	-2.81
heat shock protein family A (Hsp70) member 5	HSPA5	P20029	Cytoplasm	-1.72	-3.09
glutathione S-transferase mu 5	GSTM5	P10649	Cytoplasm	-1.63	-3.24
CD36 molecule	CD36	Q08857	Plasma Membrane	-2.44	-3.34
PDZ binding kinase	PBK	Q9JJ78	Cytoplasm	-2.65	-3.53
lipocalin 2	LCN2	P11672	Extracellular Space	-4.68	-3.58
plasminogen activator, urokinase	PLAU	P06869	Extracellular Space	1.99	-3.99
macrophage migration inhibitory factor	MIF	P34884	Extracellular Space	-1.72	-3.99
twinkle mtDNA helicase	TWNK	Q8CIW5	Cytoplasm	-5.60	-4.09
lipoprotein lipase	LPL	P11152	Cytoplasm	-2.13	-4.86
membrane bound O-acyltransferase domain containing 7	MBOAT7	Q8CHK3	Plasma Membrane	6.08	-6.44
myosin heavy chain 9	MYH9	Q8VDD5	Cytoplasm	-2.73	-7.70

[a]Normalised signal fold change of signal in treated groups with MNPs@SiO_2(RITC) to corresponding signal of in control group

Table S8. Ingenuity pathway analysis-based profiles of the metabolome of BV2 cells treated with MNPs@SiO_2(RITC)

Canonical pathways	-log(p-value)
TCA Cycle II (Eukaryotic)	7.75
Glycine Betaine Degradation	6.31
Folate Transformations I	6.04
Glycine Biosynthesis I	5.17
Acetyl-CoA Biosynthesis III (from Citrate)	4.9
Glycine Degradation (Creatine Biosynthesis)	4.79
Glutamate Degradation II	4.79
tRNA Charging	4.62
L-carnitine Biosynthesis	4.45
dTMP De Novo Biosynthesis	4.38
Tyrosine Degradation I	4.38
Sirtuin Signaling Pathway	4.2
Folate Polyglutamylation	4.16
Superpathway of Serine and Glycine Biosynthesis I	4.11
Ketolysis	4.11
Urea Cycle	4.06
Arginine Biosynthesis IV	3.9
Leukotriene Biosynthesis	3.9
Cysteine Biosynthesis III (mammalia)	3.52
Superpathway of Citrulline Metabolism	3.48

Table S9. The top 20 biological functions were evaluated algorithmically by Ingenuity Pathway Analysis in the metabolome of BV2 cells treated with MNPs@SiO_2(RITC)

Biological functions	p-value
Developmental Disorder	1.07×10^{12}
Hereditary Disorder	1.07×10^{12}
Metabolic Disease	1.07×10^{12}
Organismal Injury and Abnormalities	1.07×10^{12}
Endocrine System Disorders	3.09×10^{12}
Gastrointestinal Disease	3.09×10^{12}
Molecular Transport	5.65×10^{10}
Amino Acid Metabolism	3.26×10^{9}
Small Molecule Biochemistry	3.26×10^{9}
Cell Signaling	8.58×10^{9}
Vitamin and Mineral Metabolism	8.58×10^{9}
Cell-To-Cell Signaling and Interaction	6.14×10^{8}
Cellular Growth and Proliferation	6.14×10^{8}
Nervous System Development and Function	6.14×10^{8}
Free Radical Scavenging	8.36×10^{8}
Immunological Disease	3.3×10^{7}
Inflammatory Disease	3.3×10^{7}
Inflammatory Response	3.3×10^{7}
Neurological Disease	3.3×10^{7}
Lipid Metabolism	6.27×10^{7}

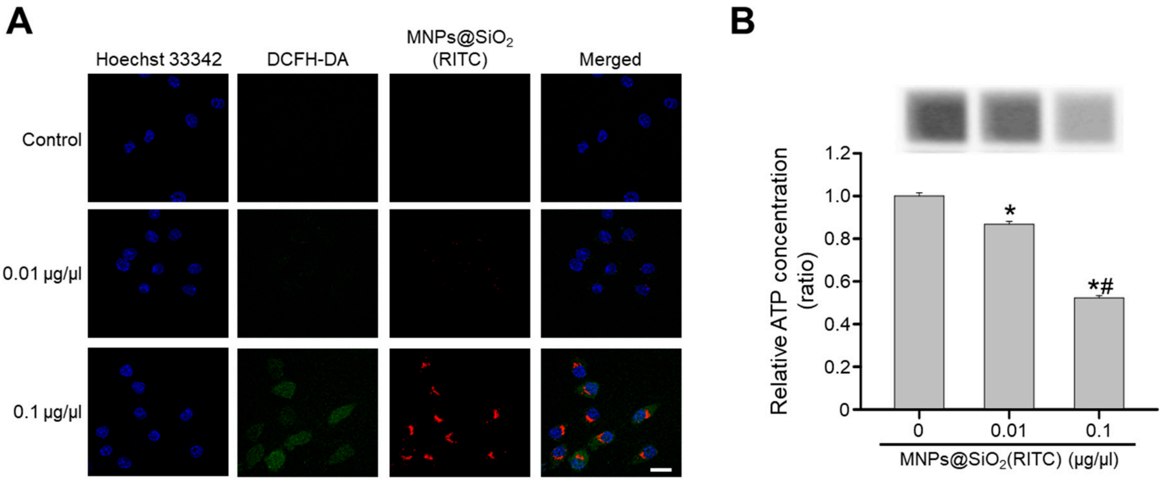

Figure S1. Generation of intracellular ROS and decrease in intracellular ATP levels in MNPs@SiO_2(RITC)-treated BV2 cells. (A) Intracellular ROS synthesis in MNPs@SiO_2(RITC)-treated BV2 cells. The cells were treated with MNPs@SiO_2(RITC) for 12 h, and ROS levels were detected by examining the fluorescence of 2',7'-dichlorodihydrofluorescein (DCF) in BV2 cells. Scale bar = 10 µm. (B) Intracellular ATP levels in MNPs@SiO_2(RITC)-treated BV2 cells. *$p < 0.05$ vs. control and $^{\#}p < 0.05$ vs. 0.01 µg/µl MNPs@SiO_2(RITC)–treated cells.

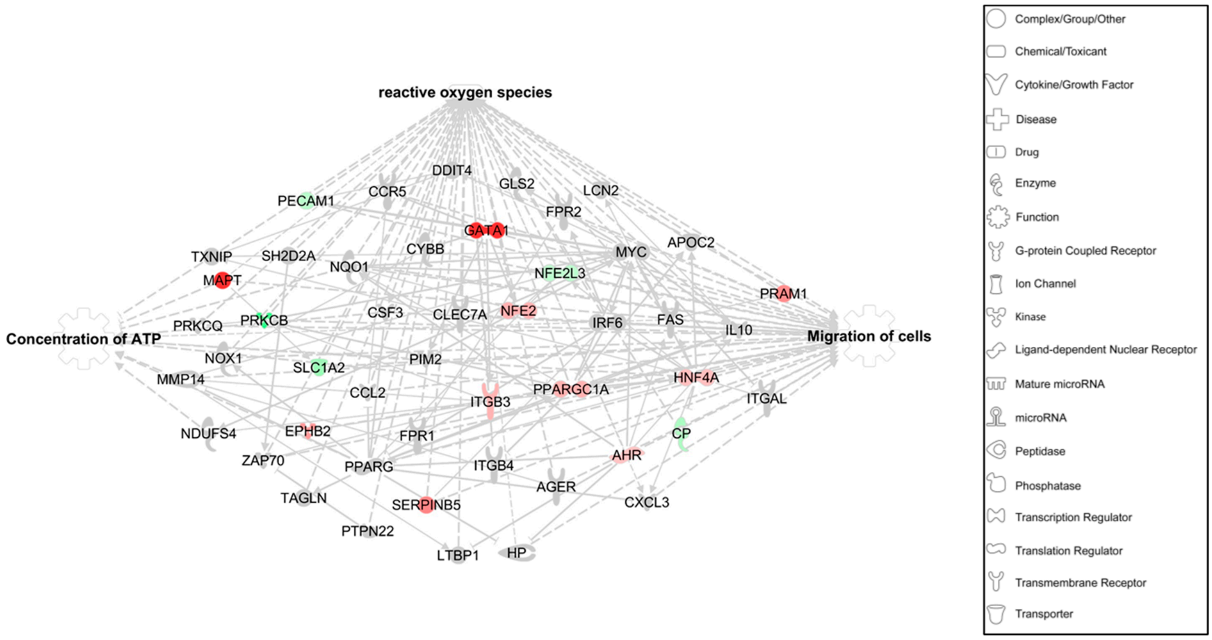

Figure S2. Functional analysis of the transcriptomic network of 0.01 μg/μl MNPs@SiO_2(RITC)-treated BV2 cells using IPA. A fold change of ± 1.5 was used as the cut-off value. Red and green areas indicate genes whose expressions were upregulated and downregulated, respectively. Path designer shapes originated from ingenuity Systems (http://www.ingenuity.com).

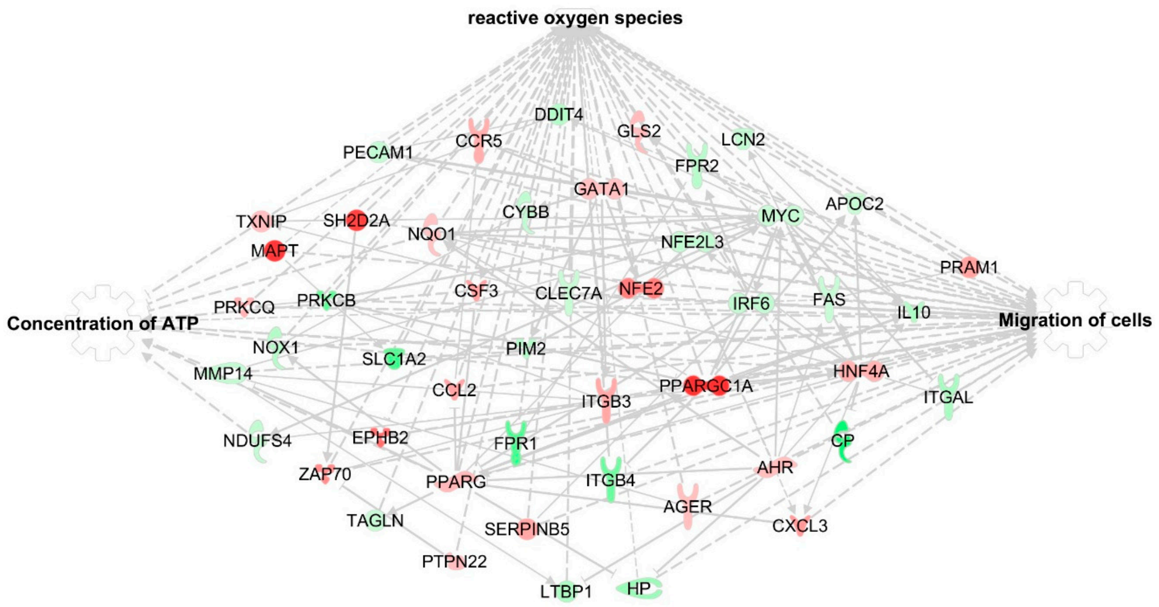

Figure S3. Functional analysis of the transcriptomic network of 0.1 µg/µl MNPs@SiO_2(RITC)-treated BV2 cells. A fold change of ± 1.5 was used as the cut-off value. Red and green areas indicate genes whose expressions were upregulated and downregulated, respectively. The symbols are shown in the legend of Figure S2.

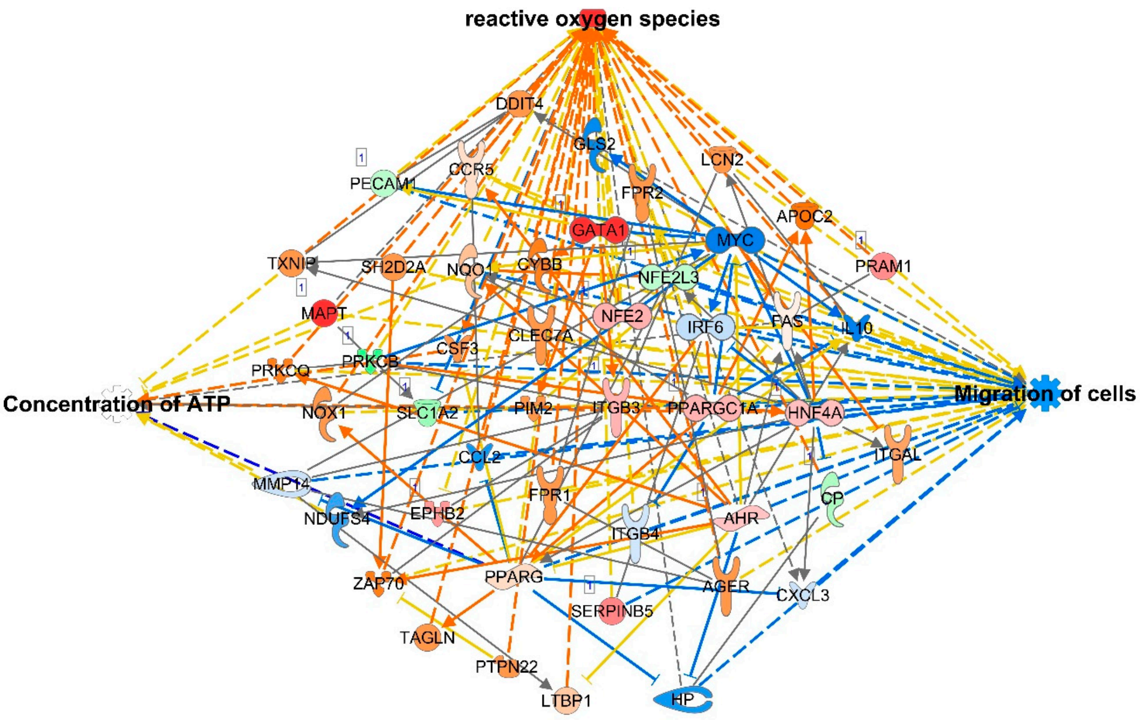

Figure S4. Functional analysis of the transcriptomic network with prediction using IPA in 0.01 μg/μl MNPs@SiO2(RITC)-treated BV2 cells. The analysis involved a fold change cut-off value of ± 1.5. Red and green nodes indicate genes whose expressions were upregulated and downregulated, respectively, compared to those of the control. The orange and blue arrows indicate the prediction of activation and inhibition, respectively. Details of shape and colour originating from IPA.

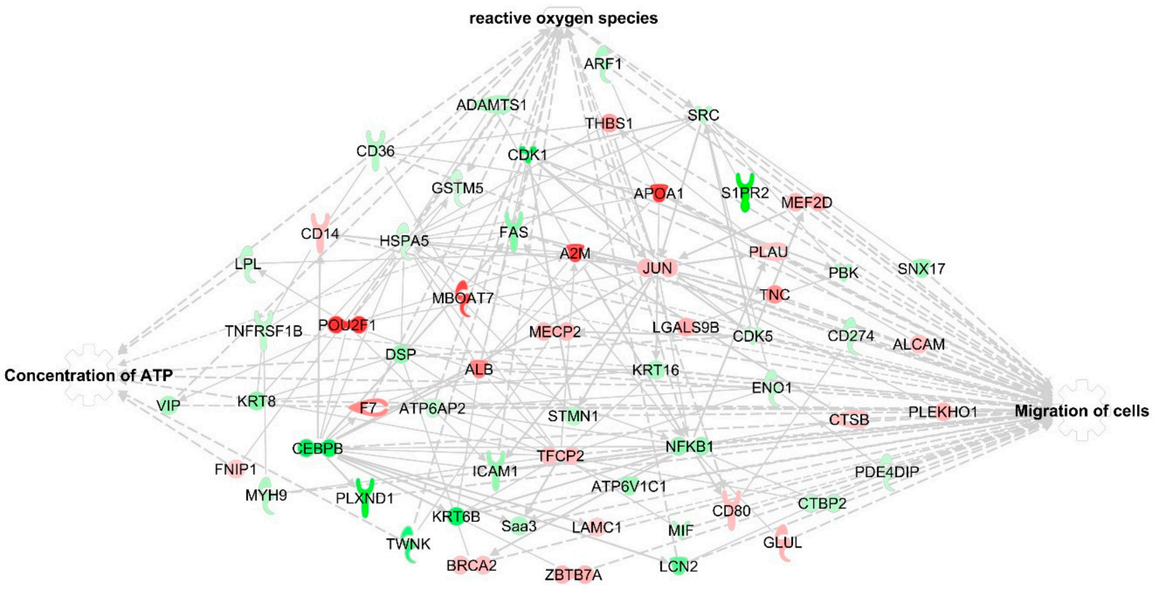

Figure S5. Functional analysis of the proteomic network of 0.01 μg/μl MNPs@SiO_2(RITC)-treated BV2 cells. A fold change of ± 1.5 was used as the cut-off value. Red and green areas indicate proteins whose expressions were upregulated and downregulated, respectively. The symbols are shown in the legend of Figure S2.

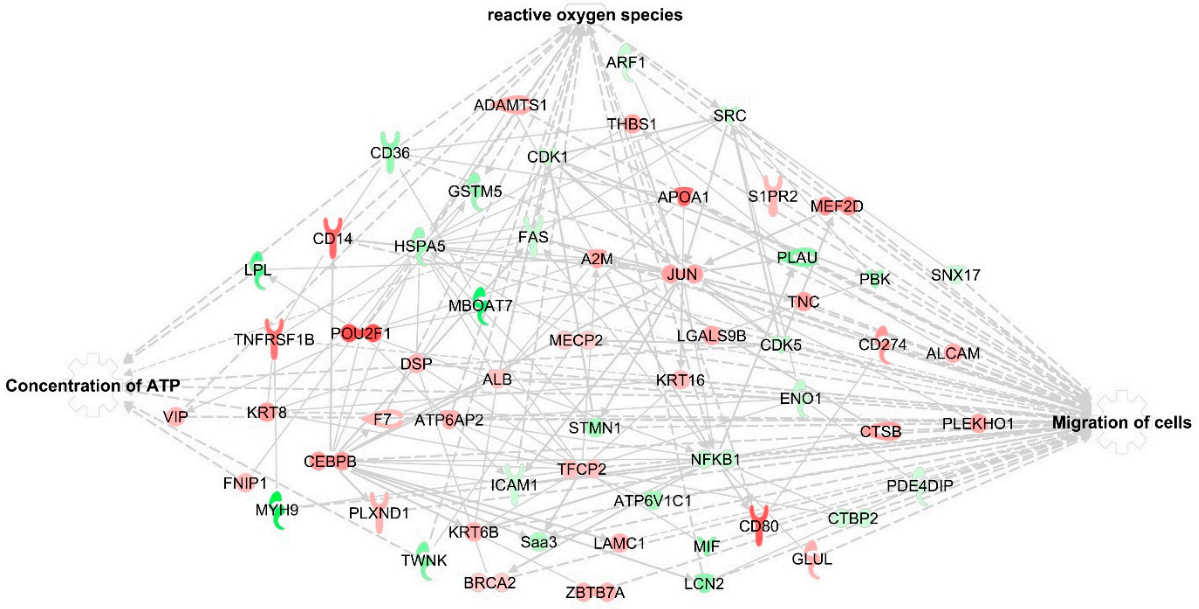

Figure S6. Functional analysis of the proteomic network of 0.1 μg/μl MNPs@SiO_2(RITC)-treated BV2 cells. A fold change of ± 1.5 was used as the cut-off value. Red and green areas indicate proteins whose expressions were upregulated and downregulated, respectively. The symbols are shown in the legend of Figure S2.

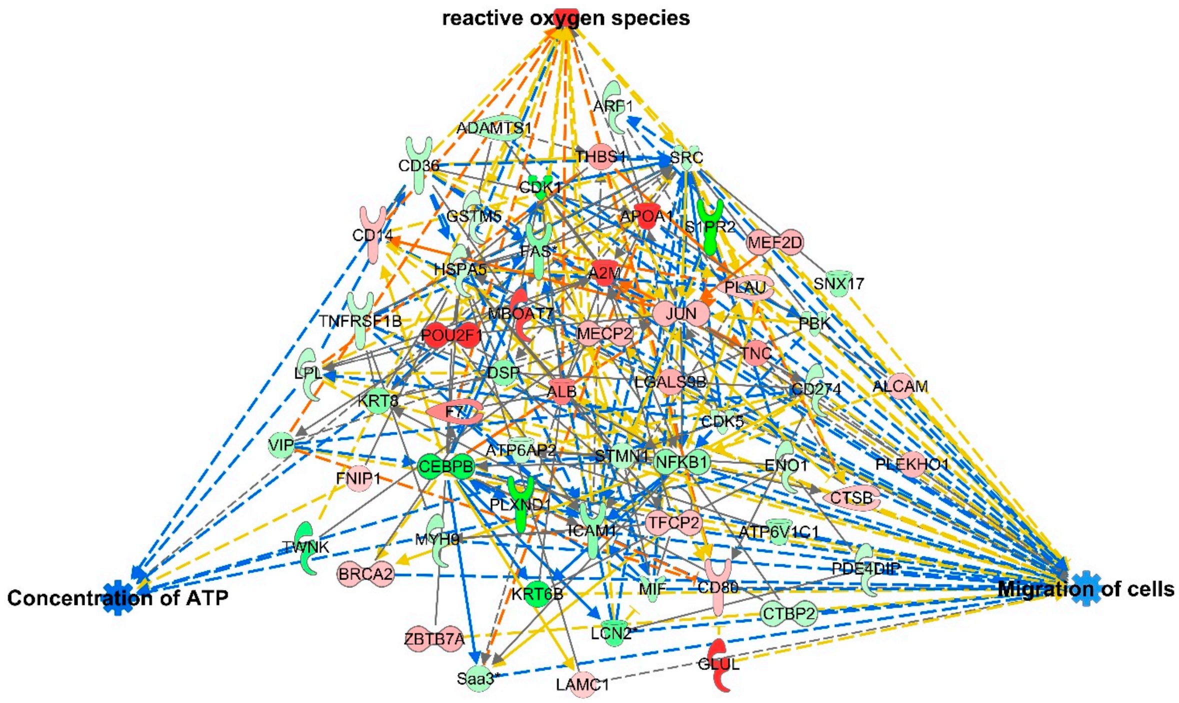

Figure S7. Functional analysis of the proteomic network in 0.01 μg/μl MNPs@SiO_2(RITC)-treated BV2 cells with prediction using IPA. The analysis involved a fold change cut-off value of ± 1.5. Red and green nodes indicate genes whose expression was upregulated and downregulated, respectively, compared to that of the control. The orange and blue arrows indicate the prediction of activation and inhibition, respectively. Details of shape and colour originated from IPA.

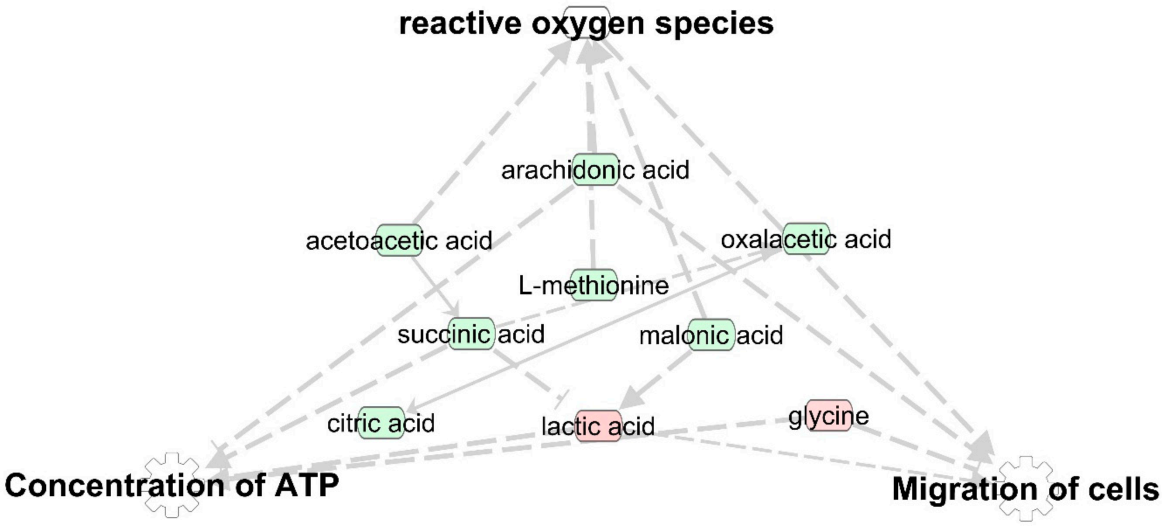

Figure S8. Functional analysis of the metabolic network of 0.01 and 0.1 μg/μl MNPs@SiO_2(RITC)-treated BV2 cells. A fold change of ± 1.2 was used as the cut-off value. Red and green areas indicate metabolites whose levels were up- and downregulated, respectively. The symbols are shown in the legend of Figure S2.

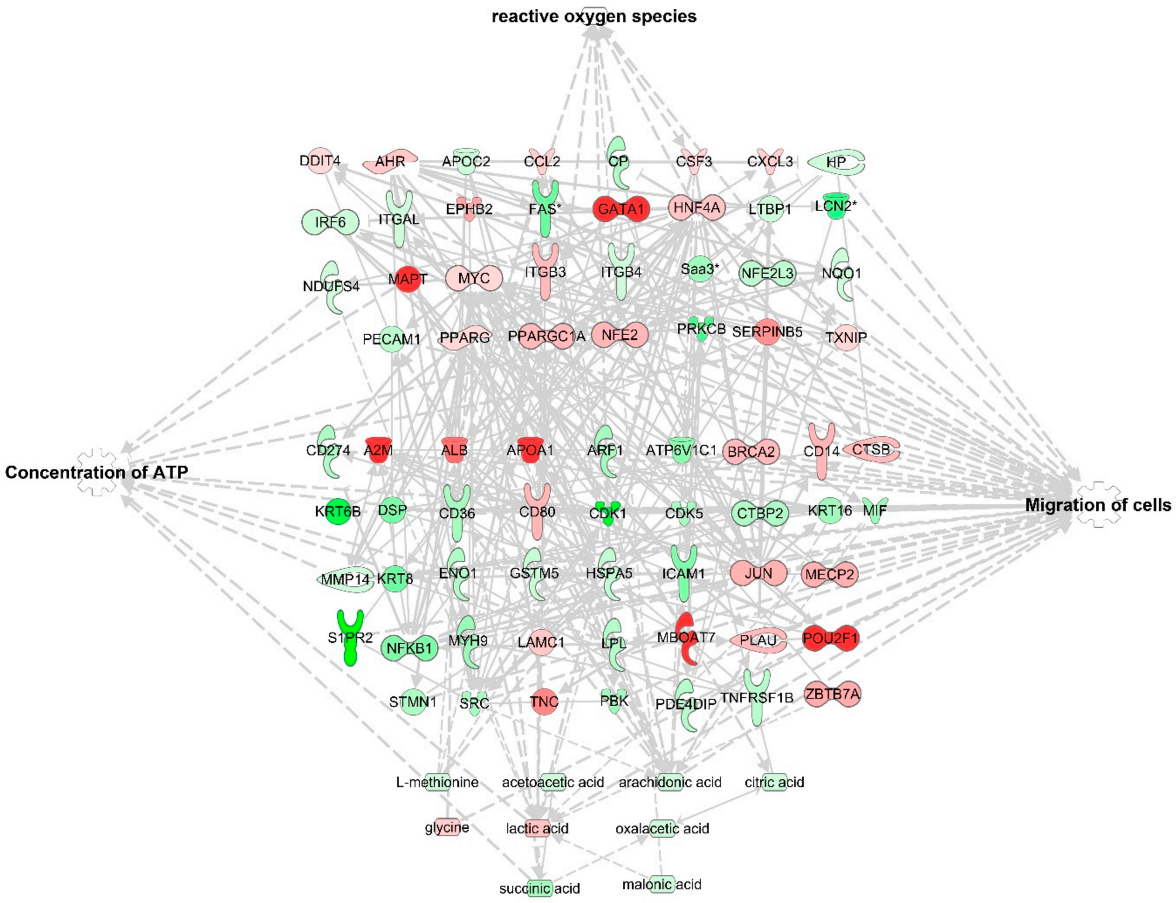

Figure S9. The merged and trimmed triple-omics network of 0.1 μg/μl MNPs@SiO_2(RITC)-treated BV2 cells. Top group: transcriptome; Middle group: proteome; Bottom group: metabolome. Downregulated Fas, Lcn2, Saa3, and Mmp14 were shared between the transcriptome and proteome. The symbols are shown in the legend of Figure S2.

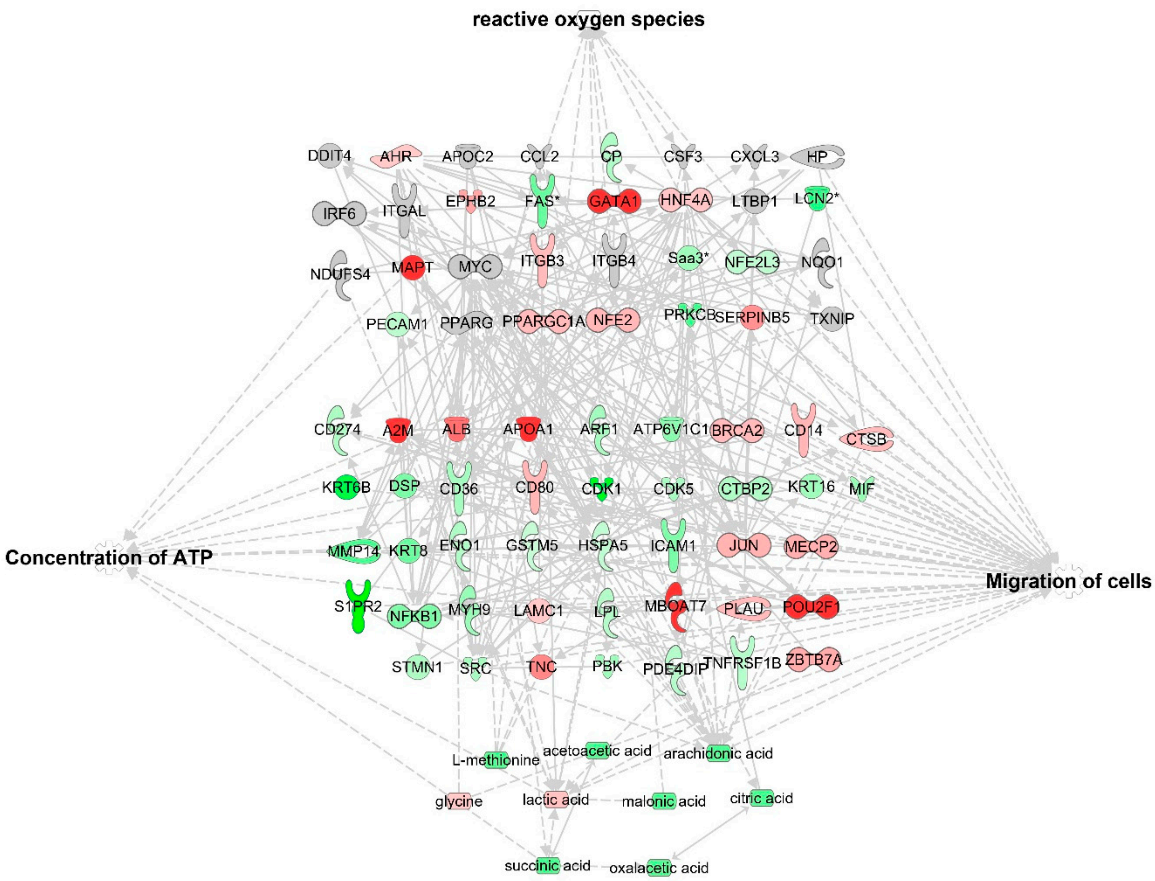

Figure S10. The merged and trimmed triple-omics network of 0.01 μg/μl MNPs@SiO_2(RITC)-treated BV2 cells. Top group: transcriptome; Middle group: proteome; Bottom group: metabolome. Downregulated Fas, Lcn2, Saa3, and Mmp14 were shared between the transcriptome and proteome. The symbols are shown in the legend of Figure S2.

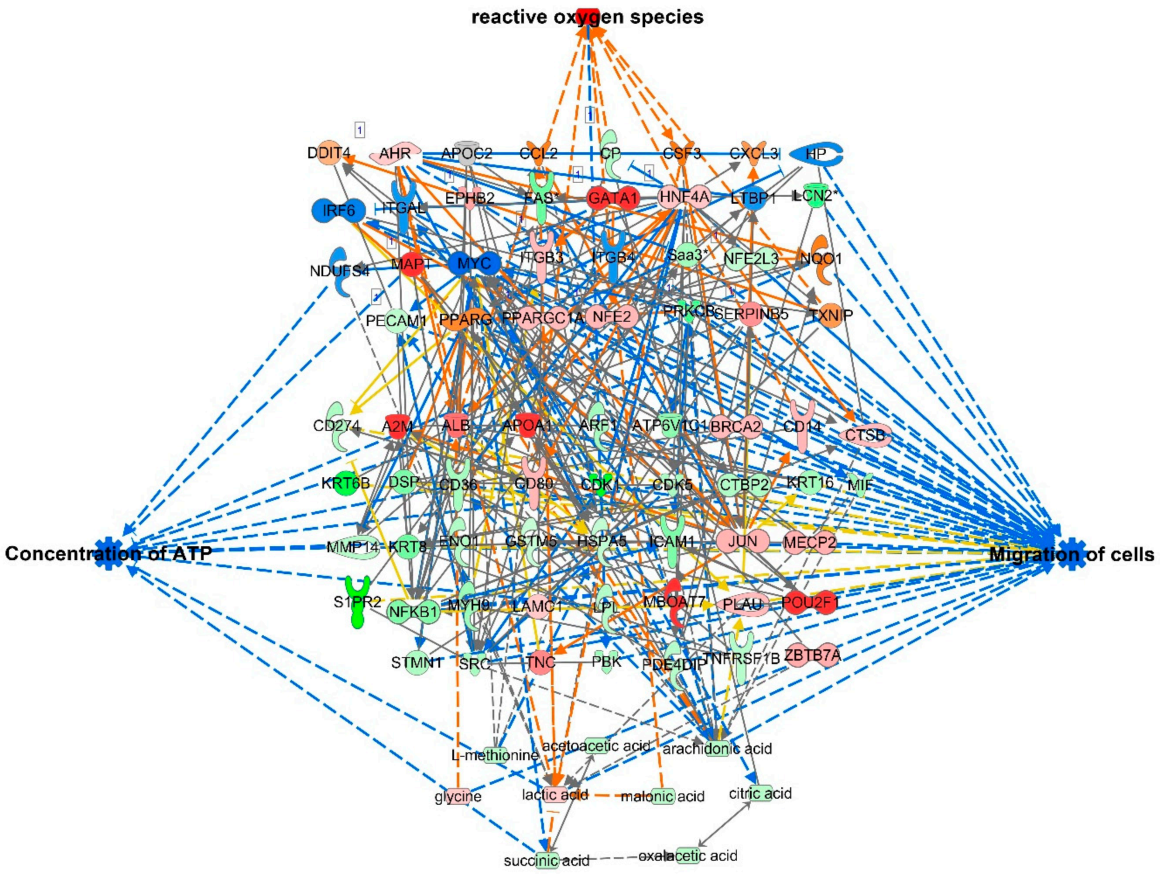

Figure S11. The merged and trimmed triple-omics network with prediction of 0.01 μg/μl MNPs@SiO2(RITC)-treated BV2 cells. Top group: transcriptome; Middle group: proteome; Bottom group: metabolome. The orange and blue areas indicate activation and inhibition, respectively. Downregulated Fas, Lcn2, Saa3, and Mmp14 were shared between the transcriptome and proteome. The symbols are shown in the legend of Figure S2.

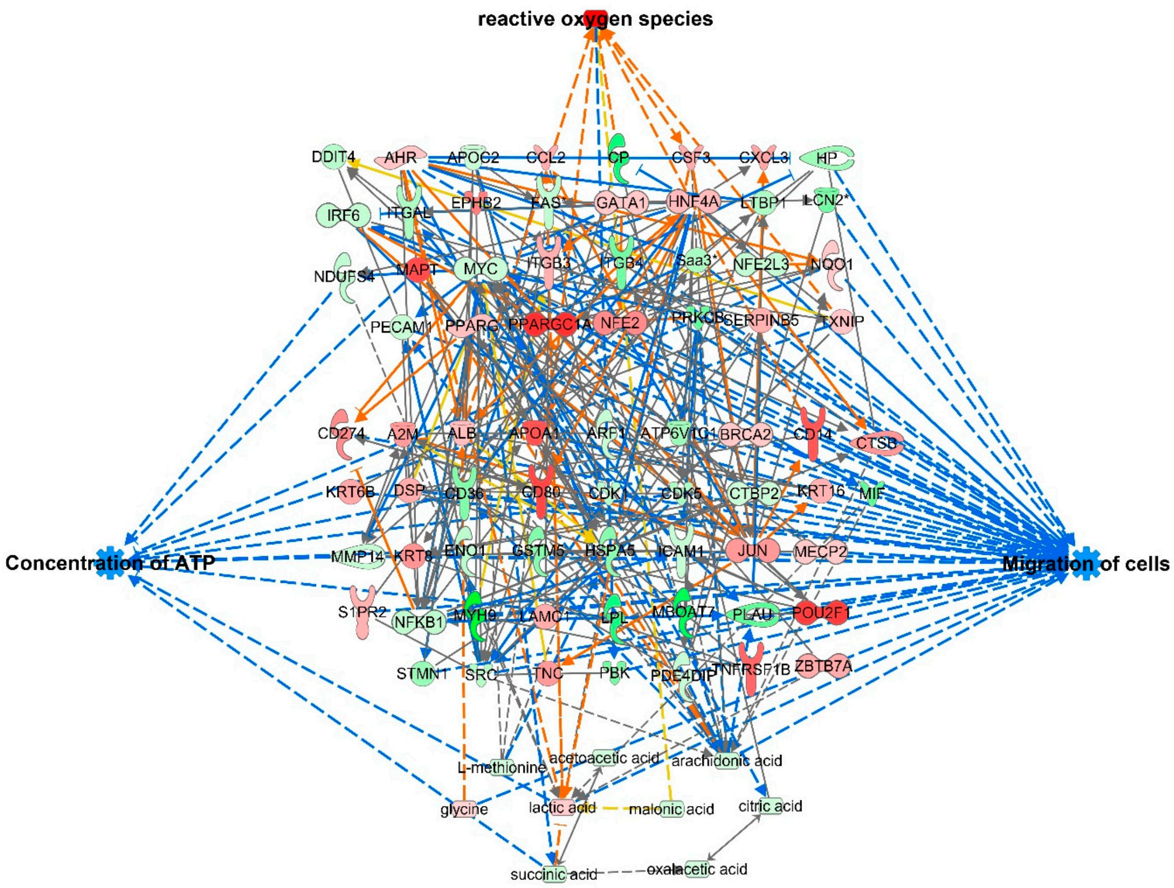

Figure S12. The merged and trimmed triple-omics network with the prediction of 0.1 μg/μl MNPs@SiO$_2$(RITC)-treated BV2 cells. Top group: transcriptome; Middle group: proteome; Bottom group: metabolome. The orange and blue areas indicate activation and inhibition, respectively. Downregulated Fas, Lcn2, Saa3, and Mmp14 were shared between the transcriptome and proteome. The symbols are shown in the legend of Figure S2.

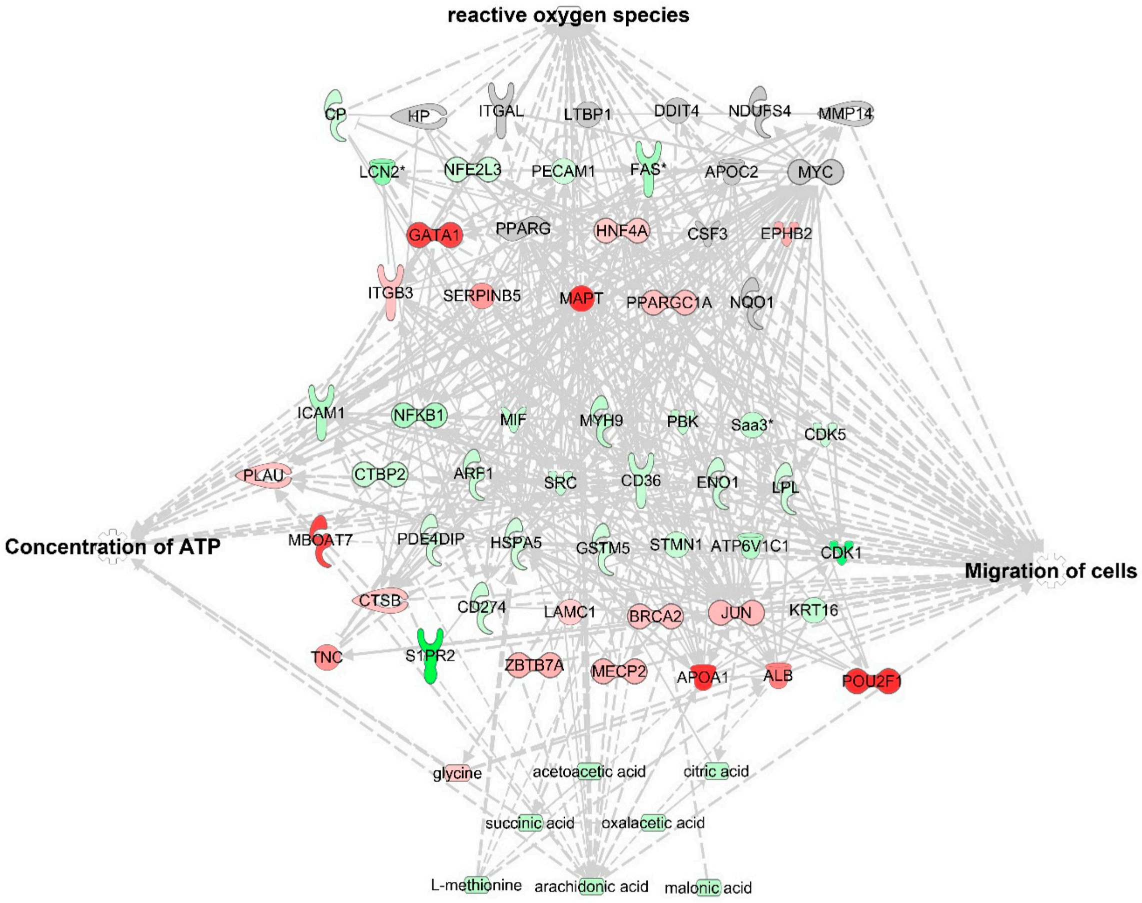

Figure S13. The trimmed network derived using a machine learning algorithm and integrated network of four categories of biological functions of omics data from 0.01 μg/μl MNPs@SiO_2(RITC)-treated BV2 cells. Top group: transcriptome; Middle group: proteome; Bottom group: metabolome. The symbols are shown in the legend of Figure S2.

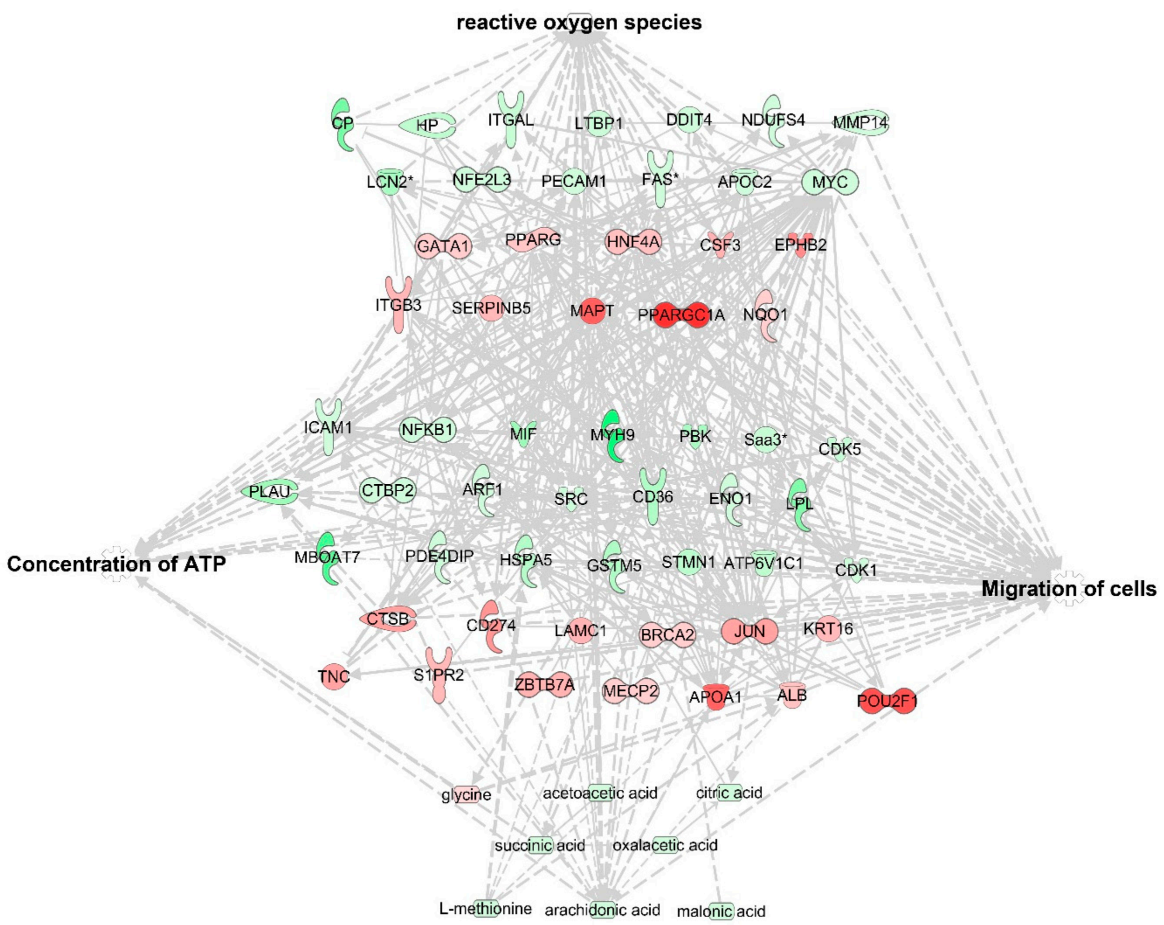

Figure S14. The trimmed network using machine learning algorithm triple-omics network of 0.1 μg/μl MNPs@SiO_2(RITC)-treated BV2 cells. Top group: transcriptome; Middle group: proteome; Bottom group: metabolome. The symbols are shown in the legend of Figure S2.

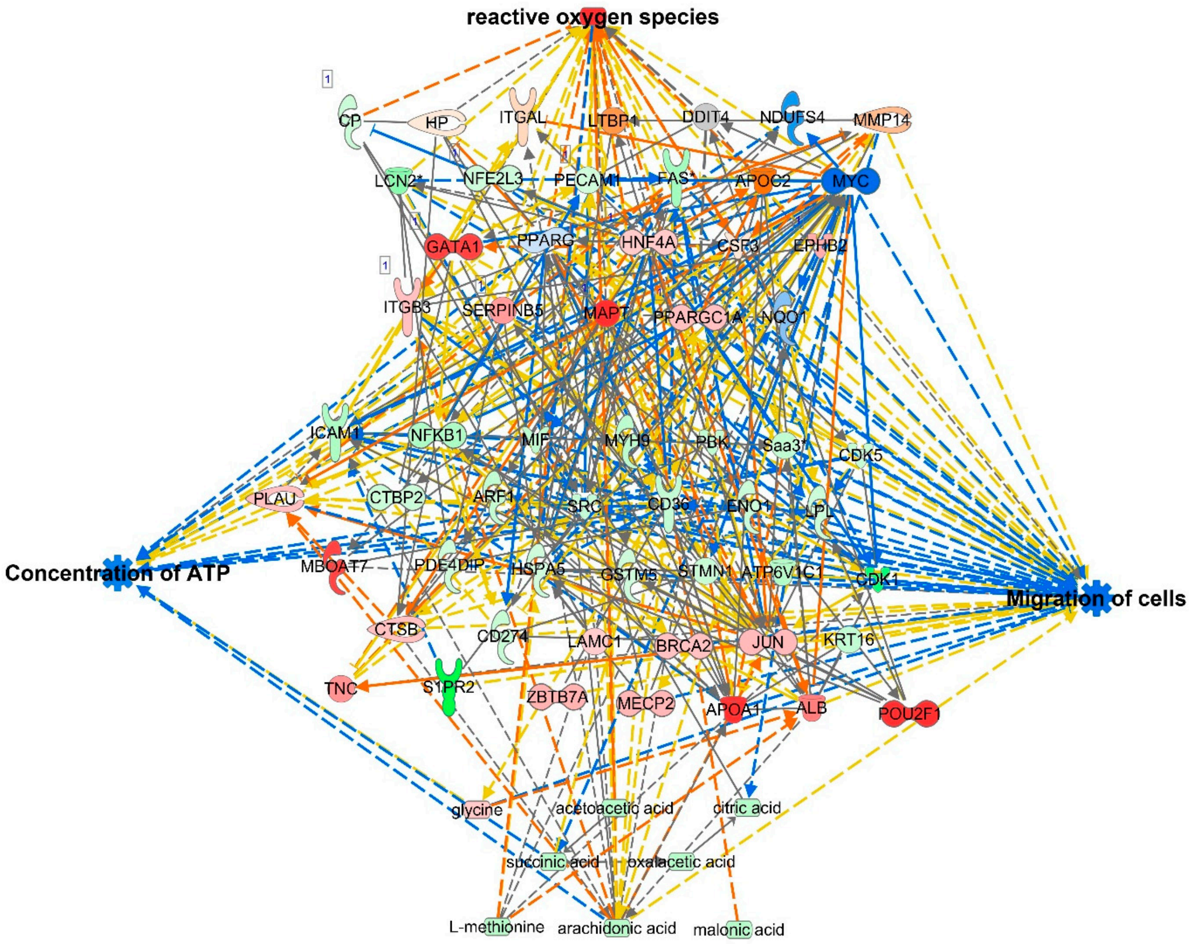

Figure S15. The trimmed network using machine learning algorithm and integrated prediction network for four categories of biological functions of omics data from 0.01 μg/μl MNPs@SiO_2(RITC)-treated BV2 cells. Top group: transcriptome; Middle group: proteome; Bottom group: metabolome. The orange and blue areas indicate activation and inhibition, respectively. The symbols are shown in the legend of Figure S2.

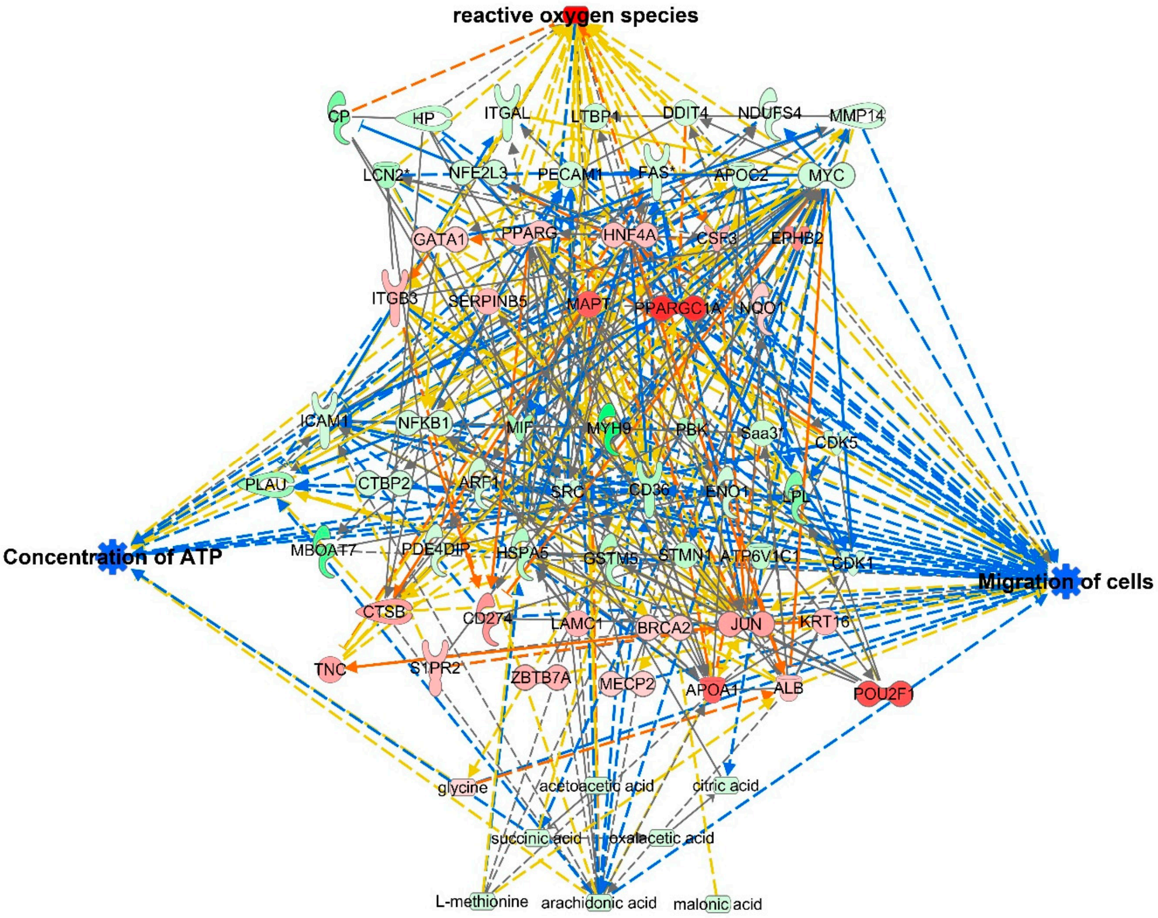

Figure S16. The trimmed network derived using machine learning algorithm and integrated prediction network for four categories of biological functions of omics data from 0.1 μg/μl MNPs@SiO_2(RITC)-treated BV2 cells. Top group: transcriptome; Middle group: proteome; Bottom group: metabolome. The orange and blue areas indicate activation and inhibition, respectively. The symbols are shown in the legend of Figure S2.

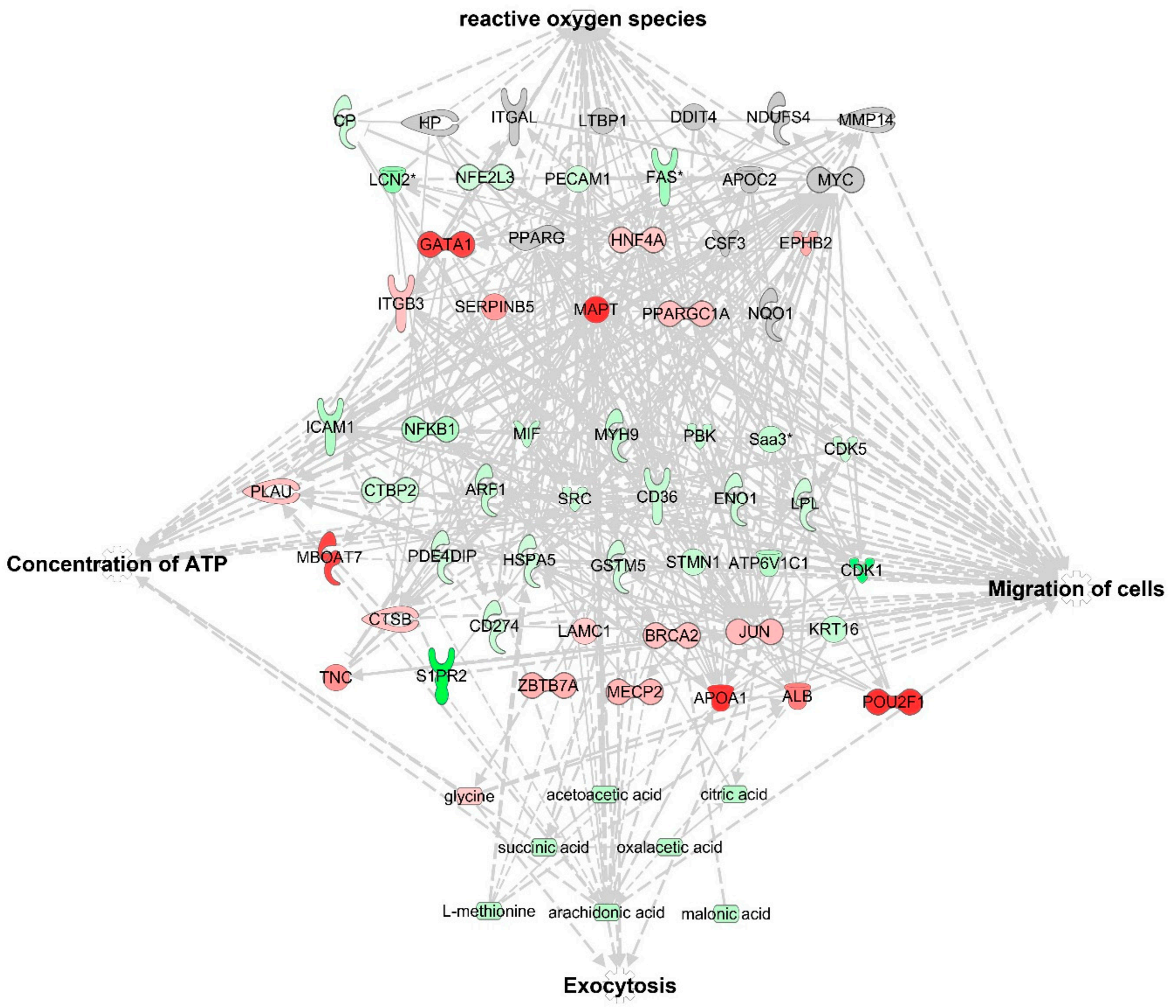

Figure S17. Trimmed network using machine learning algorithm and triple-omics network of 0.01 μg/μl MNPs@SiO_2(RITC)-treated BV2 cells, including exocytosis. Top group: transcriptome; Middle group: proteome; Bottom group: metabolome. The symbols are shown in the legend of Figure S2.

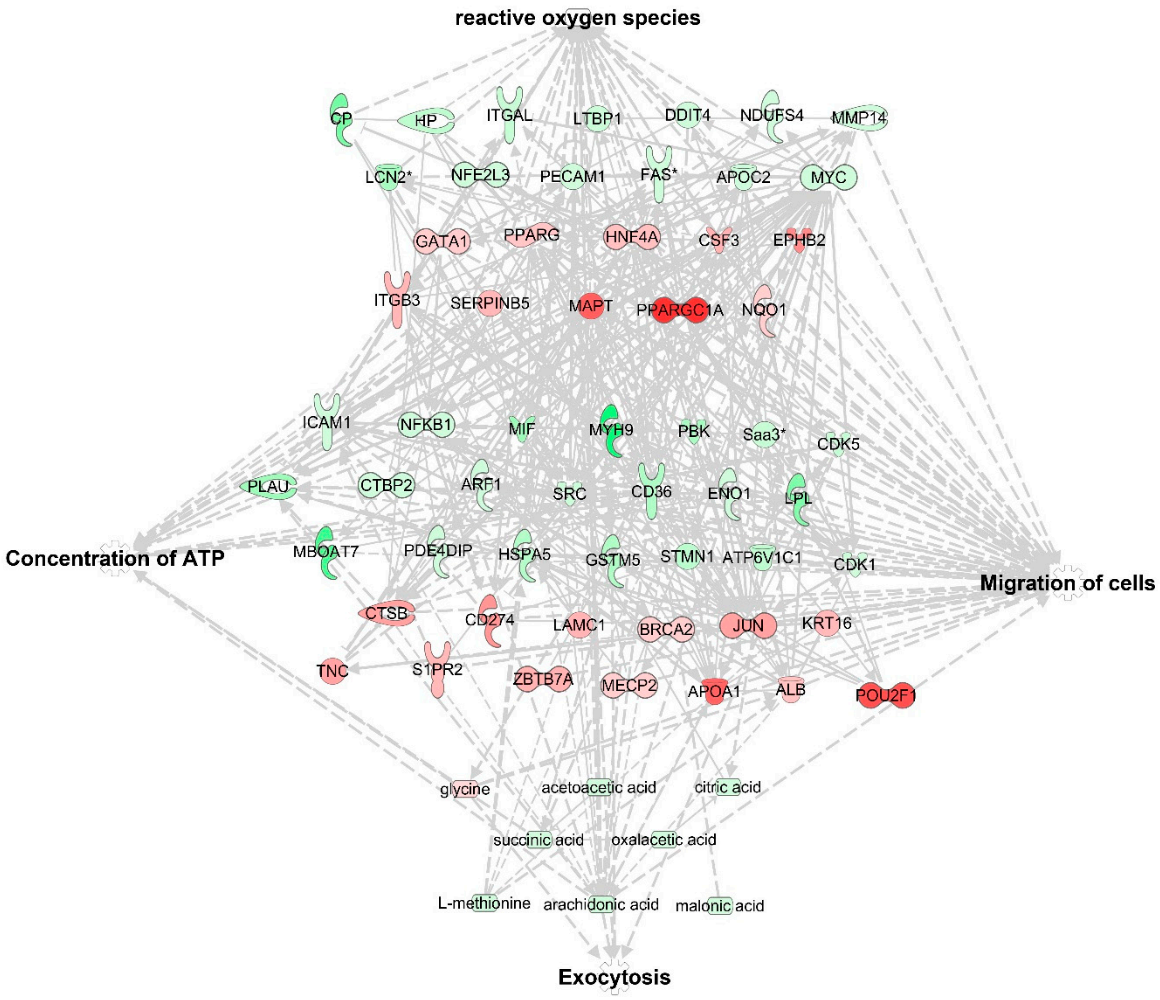

Figure S18. Trimmed network using machine learning algorithm and triple-omics network of 0.1 μg/μl MNPs@SiO_2(RITC)-treated BV2 cells, including exocytosis. Top group: transcriptome; Middle group: proteome; Bottom group: metabolome. The symbols are shown in the legend of Figure S2.

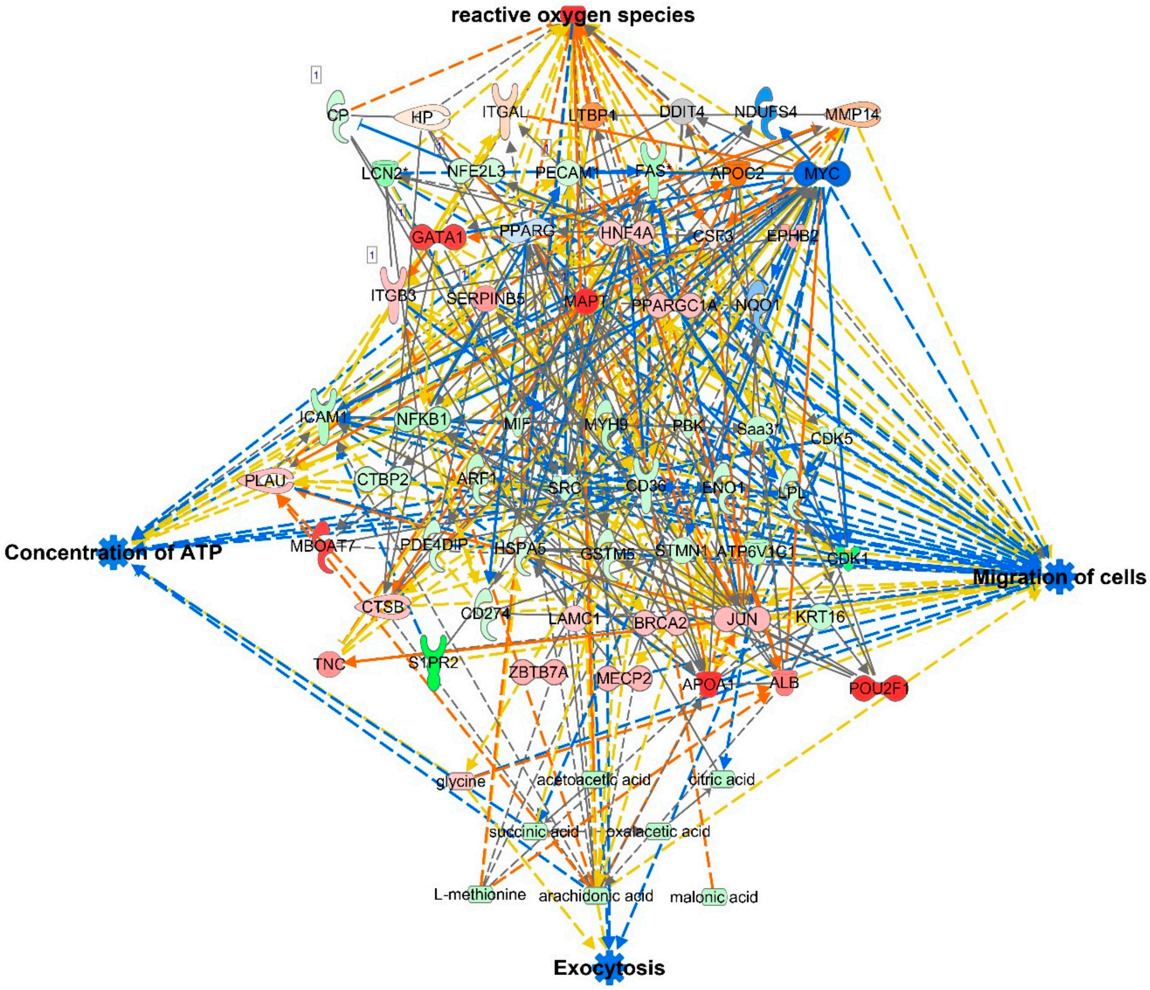

Figure S19. Trimmed network using a triple-omics machine learning algorithm with the prediction of 0.01 μg/μl MNPs@SiO_2(RITC)-treated BV2 cells, including exocytosis. Top group: transcriptome; Middle group: proteome; Bottom group: metabolome. The orange and blue areas indicate activation and inhibition, respectively. The symbols are shown in the legend of Figure S2.

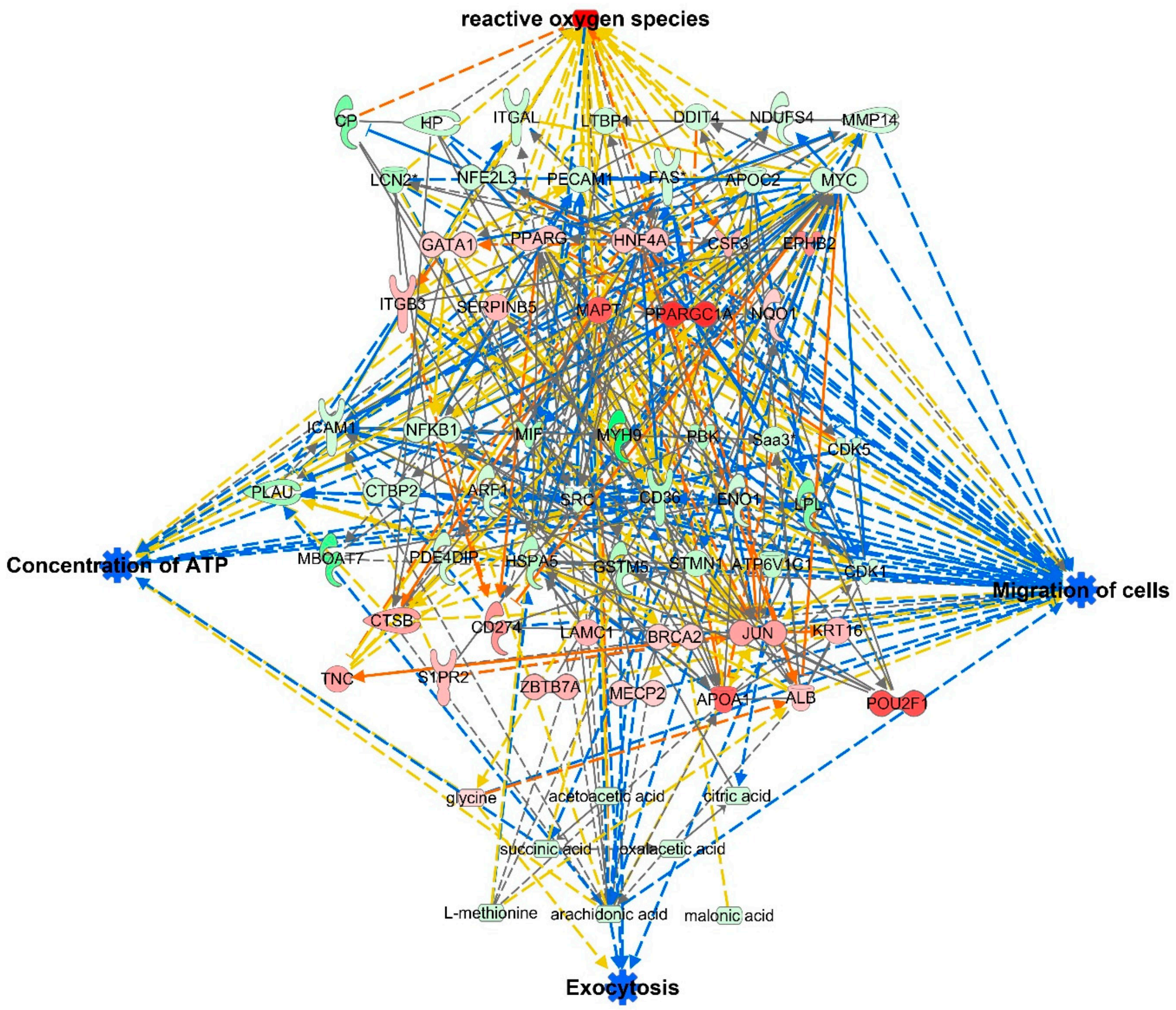

Figure S20. Trimmed network using a triple-omics network machine learning algorithm with the prediction of 0.1 μg/μl MNPs@SiO_2(RITC)-treated BV2 cells including exocytosis. Top group: transcriptome; Middle group: proteome; Bottom group: metabolome. The orange and blue areas indicate activation and inhibition, respectively. The symbols are shown in the legend of Figure S2.

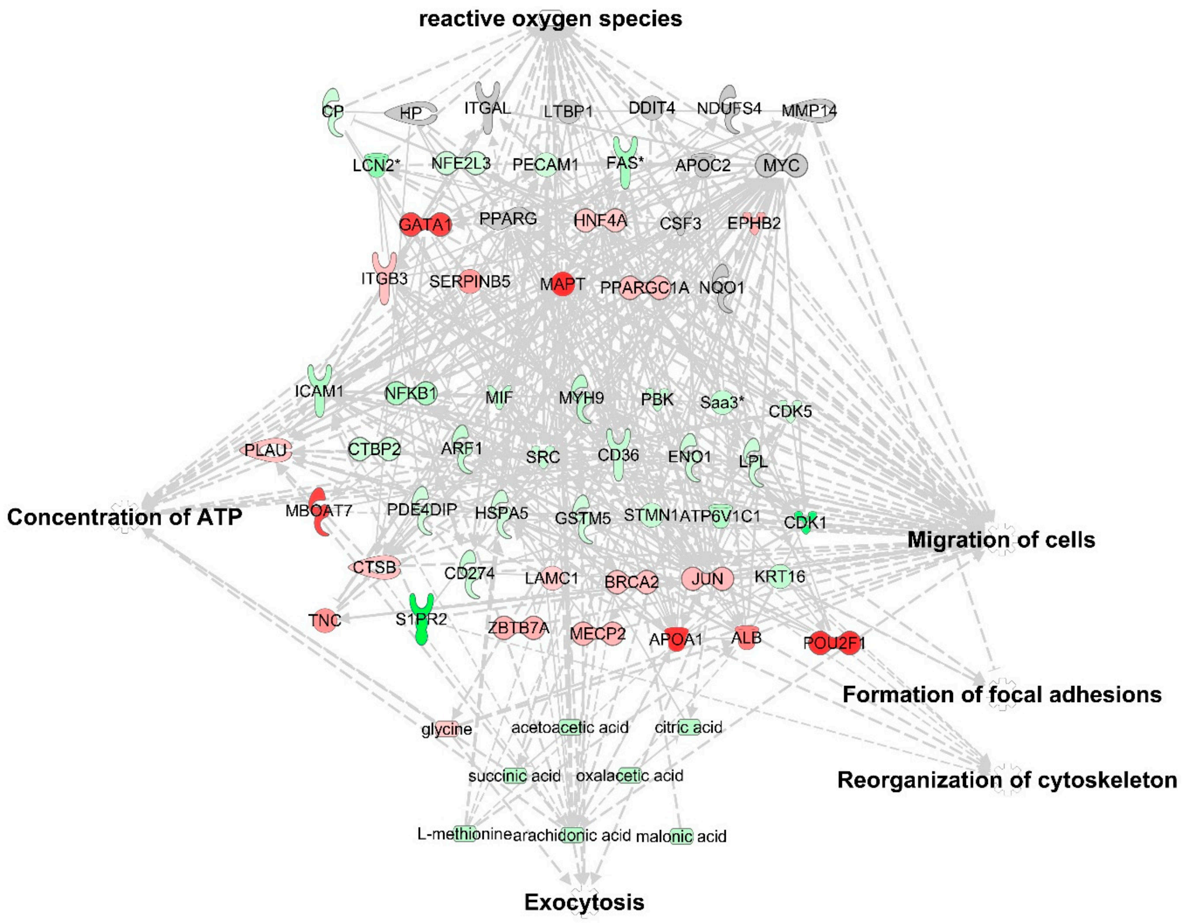

Figure S21. Trimmed network using machine learning algorithm and triple-omics network of 0.01 μg/μl MNPs@SiO_2(RITC)-treated BV2 cells, including exocytosis, formation of focal adhesions, and reorganization of cytoskeleton. Top group: transcriptome; Middle group: proteome; Bottom group: metabolome. The symbols are shown in the legend of Figure S2.

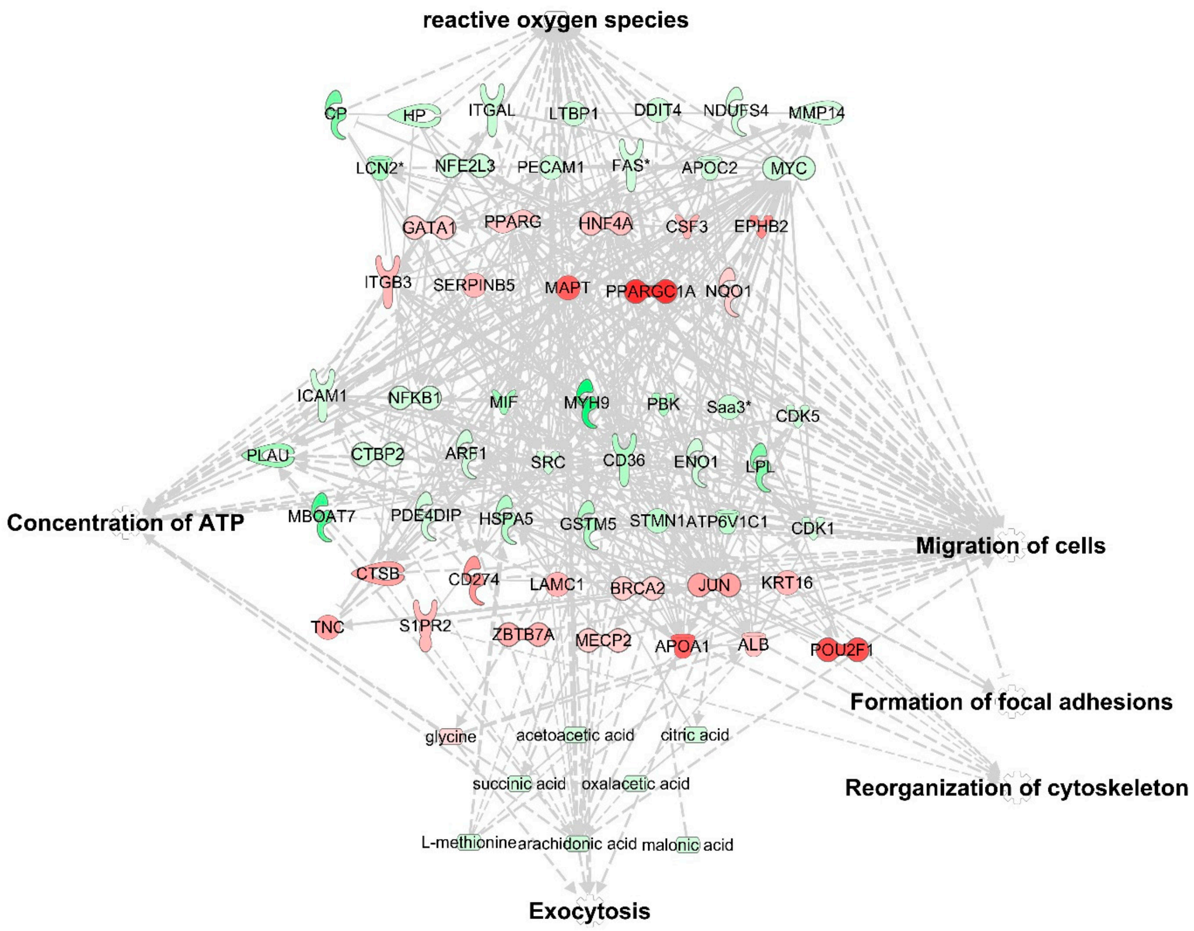

Figure S22. Trimmed network using machine learning algorithm and triple-omics network of 0.1 μg/μl MNPs@SiO_2(RITC)-treated BV2 cells, including exocytosis, formation of focal adhesions, and reorganization of cytoskeleton. Top group: transcriptome; Middle group: proteome; Bottom group: metabolome. The symbols are shown in the legend of Figure S2.

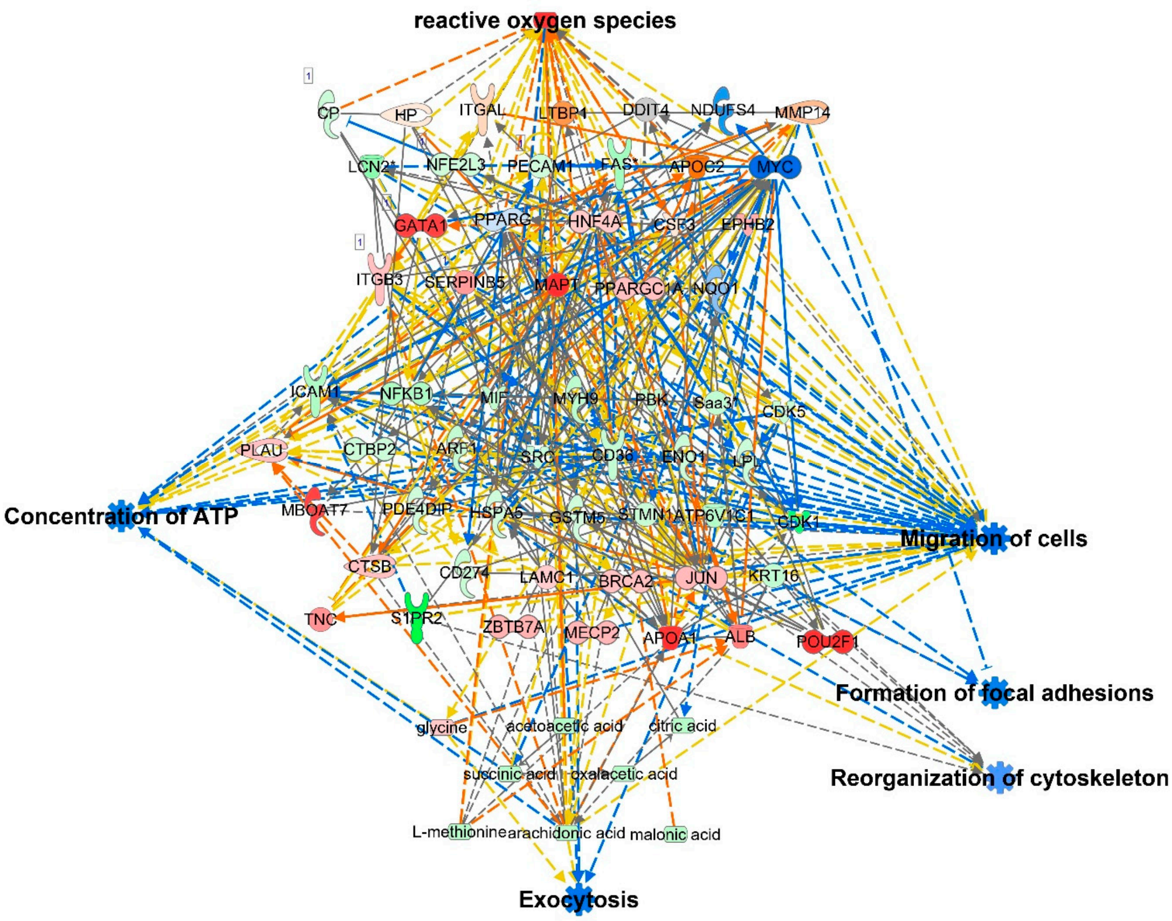

Figure S23. Trimmed network using a triple-omics machine learning algorithm with the prediction of 0.01 μg/μl MNPs@SiO_2(RITC)-treated BV2 cells, including exocytosis, formation of focal adhesions, and reorganization of cytoskeleton. Top group: transcriptome; Middle group: proteome; Bottom group: metabolome. The orange and blue areas indicate activation and inhibition, respectively. The symbols are shown in the legend of Figure S2.

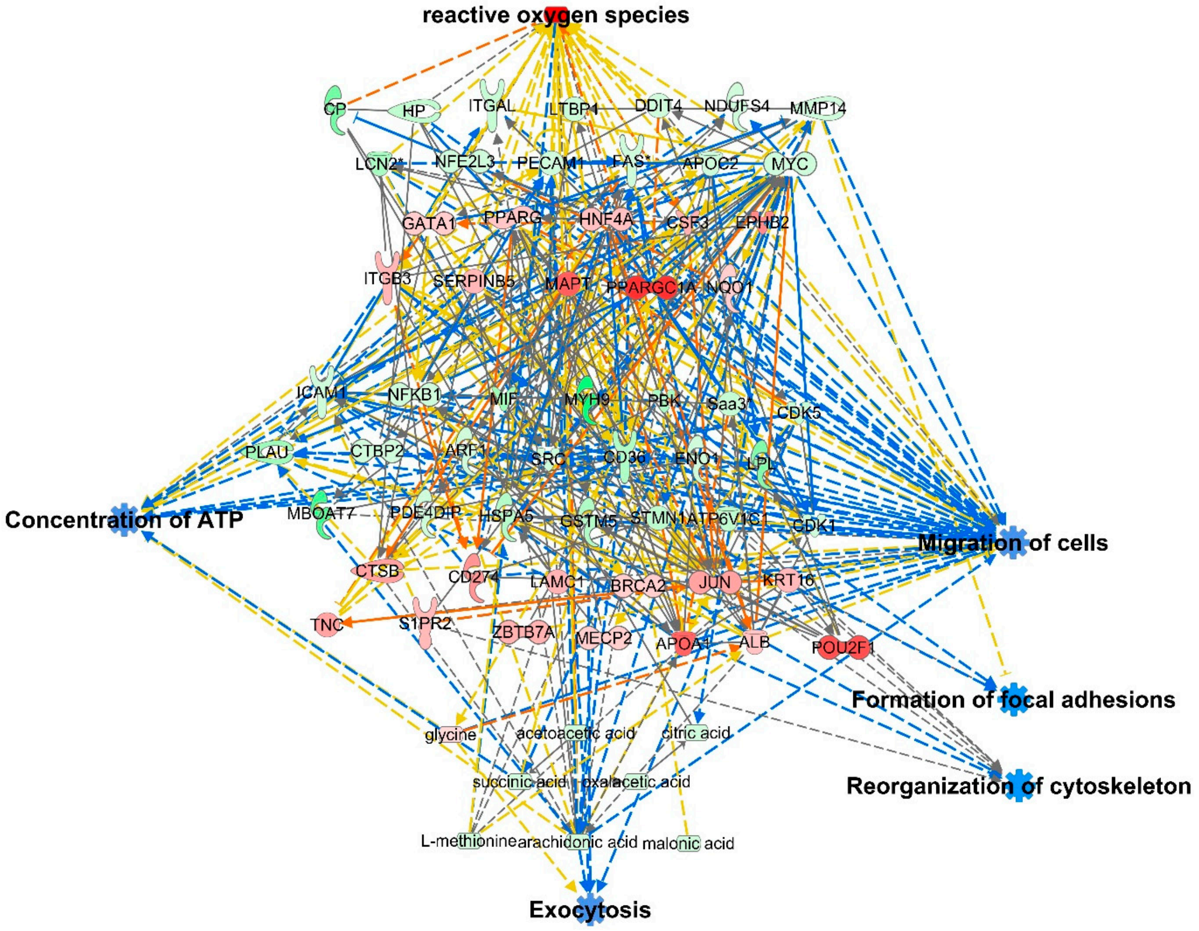

Figure S24. Trimmed network using a triple-omics network machine learning algorithm with the prediction of 0.1 μg/μl MNPs@SiO_2(RITC)-treated BV2 cells including exocytosis, formation of focal adhesions, and reorganization of cytoskeleton. Top group: transcriptome; Middle group: proteome; Bottom group: metabolome. The orange and blue areas indicate activation and inhibition, respectively. The symbols are shown in the legend of Figure S2.
